# Improved Litter Size in Thin-Tailed Indonesian Sheep Through Analysis of TGIF1 Gene Polymorphisms

**DOI:** 10.1155/vmi/7778088

**Published:** 2025-04-21

**Authors:** Mutasem Abuzahra, Dwi Wijayanti, Mustofa Helmi Effendi, Imam Mustofa, Jean Pierre Munyaneza, Ikechukwu Benjamin Moses

**Affiliations:** ^1^Doctoral Program in Veterinary Science, Faculty of Veterinary Medicine, Airlangga University, Surabaya, Indonesia; ^2^Department of Animal Science, Perjuangan University of Tasikmalaya, West Java, Tasikmalaya 46115, Indonesia; ^3^Department of Veterinary Public Health, Faculty of Veterinary Medicine, Airlangga University, Surabaya, Indonesia; ^4^Department of Veterinary Reproduction, Faculty of Veterinary Medicine, Airlangga University, Surabaya, Indonesia; ^5^Division of Animal and Dairy Science, Chungnam National University, Daejeon 34134, Republic of Korea; ^6^Department of Applied Microbiology, Faculty of Science, Ebonyi State University, Abakaliki, Nigeria

**Keywords:** genetic diversity and domesticated animal, litter size, sheep, single nucleotide polymorphism, *TGIF1*

## Abstract

Reproductive traits, particularly the litter size, are crucial for sheep husbandry. Molecular genetic selection methods, including single-nucleotide polymorphism (SNP) analyses, offer potential avenues for enhancing these traits. This study investigated the association between TGIF1 SNPs and litter size in thin-tailed Indonesian sheep. A total of 47 sheep were sampled, and their genomic DNA was analyzed. Bioinformatics, sequencing, and statistical analyses were conducted to identify SNPs, assess genetic parameters, and examine their association with litter size. Nine SNPs, including nonsynonymous variants, were successfully identified through targeted sequencing and Sanger sequencing within exon 3 of TGIF1. Noteworthy polymorphisms at g. 42725867 G>A, g. 42725886 G>A, g. 42725932 A>C, g. 42725950 A>G, g. 42726009 G>A, g.42726036 C>T, g.42726042 A>C, g. 42726051 A>G, and g. 42726059 G>A were revealed. Genetic parameter assessments indicated moderate diversity although no significant association was observed between the TGIF1 SNPs and litter size. This lack of association highlights the potential influence of environmental factors, polygenic effects, or the need for larger sample sizes in future studies. In addition, linkage disequilibrium analysis highlighted strong interconnectivity among six of the nine TGIF1 SNPs, designating them as potential Tag SNPs. Data analysis further demonstrated that the haplotype combination of H3 and Hap 6 within the identified blocks exhibited the highest litter size. This study unveils novel TGIF1 SNPs in thin-tailed Indonesian sheep, prompting the need for additional research to unravel their functional implications and potential impacts on reproductive traits. While no significant associations were found, these findings contribute to the growing body of knowledge on genetic factors influencing litter size and underscore the need for broader investigations, including whole-genome sequencing and validation in larger populations. This investigation provides valuable insights into the genetic factors that influence litter size in this breed and lays the foundation for future genetic improvement strategies.

## 1. Introduction

Sheep husbandry is an indispensable aspect of global livestock agriculture and serves as a primary source of meat and dairy production to meet the dietary demands of the human population. Reproduction assumes a pivotal role in ensuring the enduring prosperity of this vital livestock sector, emerging as a foundational and imperative element for realizing sustainable outcomes. Litter size is a pivotal reproductive characteristic in sheep, carrying notable economic benefits [1–3]. The utilization of molecular genetics and biology has been facilitated by advancements in science and technology, thereby increasing the quantity of progeny produced [4, 5]. The identification of genes that are directly or indirectly associated with litter size has gained significant traction in recent decades [5]. Increasing the litter size is not possible using traditional breeding methods [2]. Genetic enhancement has primarily been achieved using the molecular genetic-selection methodology. The process of selecting desirable traits in sheep breeding can be enhanced through the precise identification of single nucleotide polymorphisms (SNPs). This molecular-based selection method enables the linkage between specific genes and desired qualities, resulting in increased accuracy [6]. SNP characterization is a commonly used method for detecting genetic variations across individuals. This involves identifying SNP-based polymorphisms to assess the genetic variation in the genes responsible for specific phenotypes [7].

Genetic variety is crucial for promoting the development of sophisticated genes, safeguarding existing populations, advancing evolutionary processes, and enabling adaptations to changing environmental conditions [8, 9]. Identification of gene polymorphisms is crucial for breeding farm animals. This study aimed to establish animal genotypes and their connections to productive, reproductive, and economic characteristics [10]. Furthermore, marker-assisted selection (MAS) can be employed to improve overall production efficiency and increase offspring size [5, 11].

TGIF1, which is also known as TGF-β Induced Factor Homeobox 1, has been found to help change how sensitive FSH-β is to pulses of gonadotropin-releasing hormone (GnRH) [12], which is critical for follicular development and ovulation. TGIF1 belongs to the TALE homologous domain family of proteins [13–15]. TGIF1 regulates the production and secretion of FSH through various mechanisms. In vertebrates, TGIF1 is a transcriptional repressor that inhibits SMAD-induced TGF-β signaling and interacts directly with SMAD genes; TGIF1's association with SMAD proteins, key mediators of TGF-β signaling, demonstrates its regulatory role. TGIF1 is expected to interact with SMAD2, a gene involved in reproductive functions [15]. Several studies have identified SMAD2 as a potential gene that influences reproductive features in livestock with varied fecundity [16, 17]. It is highly expressed in sheep ovaries [18] and highly conserved in mammals [12, 15]. It is also thought that TGIF1 stops the release of 17β-estradiol (E2) and progesterone (P4) in the ovarian granulosa cells (GCs) of dairy goats by stopping the activity of the transcription factor of the specificity protein 1 (SP1) [13]. Nucleotide variations in exon III of the TGIF1 gene have been observed across different sheep breeds. These variations are believed to influence reproductive responses to seasonal changes and enhance overall reproductive performance [12, 15]. On the other hand, the variation in TGIF1 related to reproductive characteristics in other species remains unexplored. These findings indicated that TGIF1 plays a crucial role in mammalian reproductive processes. Identifying SNPs and analyzing their associations with litter size are crucial steps in understanding the genetic basis of reproductive traits. Therefore, the primary objective of this investigation is to identify TGIF1 polymorphisms in thin-tailed sheep through sequencing and analyze their association with litter size.

## 2. Materials and Methods

### 2.1. Ethics Statement

The use of animals in experiments was authorized by Airlangga University's Faculty of Veterinary Medicine. This study has ethical authorization from the Animal Care and Use Committee (ACUC) (no: 1. KEH.117.09.2022).

### 2.2. Animal

A total of 47 local thin-tailed ewes were used in the current study for blood sample collection and litter size data. All the sheep were randomly selected and reared at the Berkah Animal Farm. During the study, the animals were fed a daily ration of leguminous and gramineous grasses equivalent to 10% of their body weight, supplemented with a commercial concentrate feed at 5% of their body weight per individual. The concentrate feed contained 15% crude protein, as detailed in our previous study [4]. All the sheep were female, with a parity of 1–3, and all individuals were healthy. A sample size of 47 sheep was chosen based on availability and logistical constraints. While limited for a genetic association study, it provides a preliminary basis for identifying TGIF1 polymorphisms and their potential link to litter size in thin-tailed Indonesian sheep. Larger studies are needed for validation.

### 2.3. Sample Preparation

Blood samples were aseptically collected from thin-tailed sheep ewes (*n* = 47), from the jugular vein, yielding approximately 5 mL of blood per ewe. EDTA was used as an anticoagulant during the procedure. Genomic DNA was isolated from whole blood samples using the phenol–chloroform extraction method. Subsequently, it was dissolved in TE buffer consisting of Tris HCl (10 mM) and EDTA (1 mM) at a pH of 8.0. The DNA solution was then stored at a temperature of −20°C.

### 2.4. PCR Amplification and DNA Sequencing

Using the TGIF1 reference sequence for sheep (XM_042239656.2), we designed primers for PCR amplification with NCBI Primer Blast (https://www.ncbi.nlm.nih.gov/tools/primer-blast/index.cgi). A primer set was used to amplify the exon 3 genomic region of the TGIF1 gene. Primers for the targeted region were designed using Primer3Plus software (Primer3Plus-Pick Primers) and tested for specificity in silico to ensure they bind only to the intended region before synthesis (fragment 1 F: GAAGAGAGCCCATTTCACTC and R: TCTTTTGCTATCATCCCAGC). PCR amplification was performed using a T100 Thermal Cycler (Bio-Rad) with a total reaction volume of 30 μL. The reaction mixture included 2 μL of genomic DNA, 0.7 μL each of forward and reverse primers, 12.6 μL of Taq Green Master Mix, and 14 μL of nuclease-free ddH_2_O. The thermal cycling protocol comprised an initial denaturation at 94°C for 3 min; denaturation at 94°C for 30 s; annealing at 52°C for 30 s; 37 cycles of elongation at 72°C for 1 s; and a final extension at 72°C for 5 min. The products were identified by the utilization of electrophoresis on a 1.5% agarose gel that had been treated with ethidium bromide for staining purposes. The PCR products, with a volume of 25 μL, were submitted to 1st BASE DNA for analysis via Capillary Electrophoresis. The DNA sequences were further analyzed using BioEdit ver. 7.00 (Tom Hall, Ibis Therapeutics, California, USA). The SNPs were validated through electropherogram analysis, with sequencing performed by 1st Base Company in Selangor, Malaysia, to confirm the polymorphisms.

### 2.5. Bioinformatics Analysis for Gene Conservation

TGIF1 amino acid sequences of nine species—Ovis aries (XP_042095590.1), Capra hircus (XP_017895252.1), Ovis ammon polii (KAI4532774.1), Bos mutus (XP_005893705.1), Moschus berezovskii (XP_055273130.1), Bubalus bubalis (XP_006071907.1), Bos javanicus (XP_061255833.1), Bubalus carabanensis (XP_055415199.1), Oryx dammah (XP_040120604.1), and Dama dama (XP_060987399.1) The protein sequences were obtained from the NCBI database. To determine the sequences' similarity, we used Protein Blast (https://blast.ncbi.nlm.nih.gov/Blast.cgi). For the alignment using the MUSCLE method of the MegAlign program, the phylogenetic tree was built using MEGA11 for evolutionary analysis and amino acid sequence alignment. We used STRING v11 (https://cn.string-db.org/cgi/input?sessionId=bd0GhtJmE6ee) to build the protein-protein interaction network [2].

### 2.6. Statistical Analysis

Genotyping data were calculated using Pop Gene Version 1.32 to compute allele and genotype frequencies, assess polymorphism information content (PIC), evaluate heterozygosity (HE), determine the number of effective alleles, and use chi-square (*χ*2) tests to derive the corresponding *p* value [19, 20]. The association between different SNP litter size and genotypes was determined using the least squares mean test with the following mixed linear model: *Y*_*ik*_=*μ*+*G*_*i*_+*e*_*ik*_. (*Y*_*ik*_: the phenotypic value of each litter size; *µ*: the overall average; *G*_*i*_: the fixed effect of the genotype; and *e*_*ik*_: random error) [21]. Using SPSS software (Version 25.0, International Business Machines Corporation, New York, USA). The models excluded the effects of farm and parity of ewes and the effects of male sheep, which had no significant effects on the variation of traits in this population. Statistical significance was *p* < 0.01 and *p* < 0.05. Post hoc comparisons were performed using Bonferroni adjustments within the SPSS GLM framework to control for multiple testing and reduce the likelihood of type I errors. In addition, we used Haploview4.2 [22] to investigate the structure of linkage disequilibrium (LD) and determined the haplotypes as well as the *D*′ and *r*^2^ LD. If *r*^2^ < 0.33, the LD is not sufficiently strong; if *r*^2^ > 0.33, the LD is sufficiently strong; and if *r*^2^ = 1, the LD is complete.

## 3. Results

### 3.1. SNPs Identification by Sequencing

**Figure 1:**
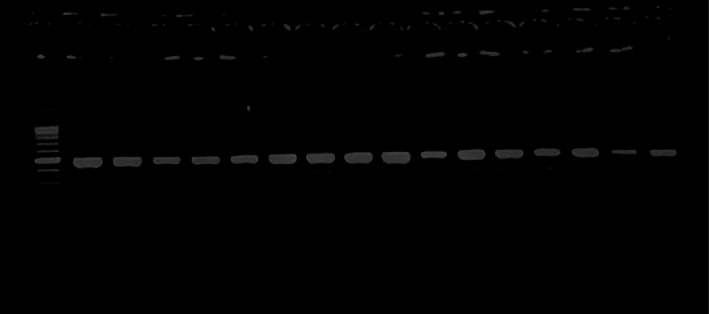
A 483 bp PCR amplification of TGIF1 gene specific fragment.

PCR products were separated into 1.5% agarose gels. The 483 bp amplified fragments matched the expected target size, confirming the robust specificity of the amplification process, as shown in (Figure 1). The sequences of the TGIF1 exon III region from the total samples were compared with the reference sequences XM_042239656.2 and XM_042235282.2 from GenBank. All genotype positions were inserted according to the sheep genome assembly version Oar_rambouillet_v3.0:23: 38,010,699:38,019,077 ARS-UI_Ramb_v3.0 (accession RefSeq GCF_016772045.2). As shown in Table 1 and Figure S1, nine SNPs were detected in the studied sheep, five nonsynonymous and four synonymous variants. Among these, five SNPs caused amino acid changes in the protein sequence: 42725867 G>A, resulting in Arg/Lys; 42725932 A>C, causing Thr/Pro; 42725950 A>G, causing Lys/Glu; 42726052 G>A, leading to Ala/Thr; and 42726055 G>A, causing Glu/Lys. Remarkably, six of the identified SNPs have not been previously described in other sheep breeds. Notably, three of these novel SNPs were nonsynonymous polymorphisms: g.42726055 G>A, causing a nonconserved change from glutamic acid to lysine (Glu217Lys) (SNP9) and g.42725932 A>C, causing Thr176Pro (SNP3). Both substitutions were predicted as deleterious by the Variant Effect Predictor (VEP) software, with SIFT values of 0.14 and 0.18, respectively. The novel SNPs may possess functional importance, as nonsynonymous mutations frequently modify protein structure or function, potentially influencing the involvement of the TGIF1 protein in reproductive processes, despite the absence of an association with litter size in the present research. The other SNPs detected in this study were silent. The SNPs numbers, locations, loci, and amino acid changes in TGIF1 are summarized in Table 1.

### 3.2. Bioinformatics Analysis

**Figure 2:**
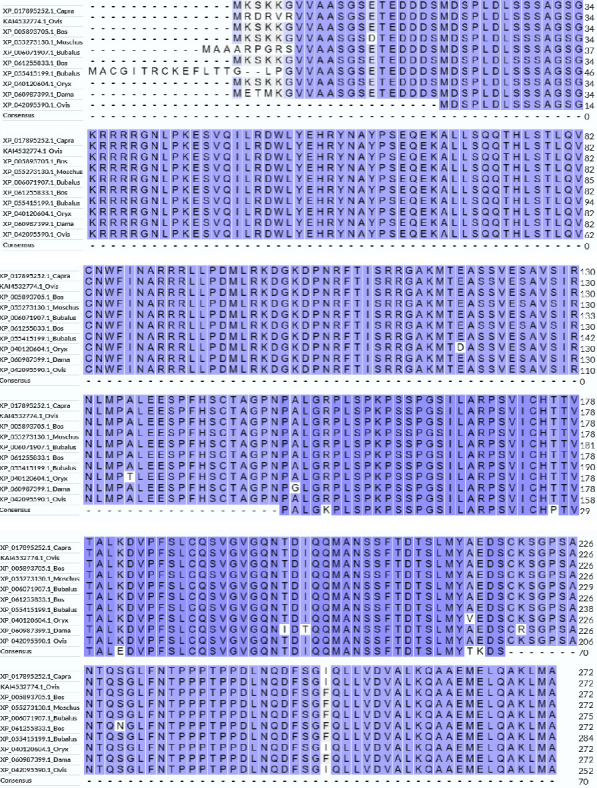
The amino acid sequence alignments of TGIF1 were performed using the MEGA11 tool, where identical residues were aligned and colored according to the consensus character assigned under each column.

Ten different species' TGIF1 amino acid sequences were obtained. Ovis aries, Capra hircus, Ovis ammon polii, Bos mutus, Moschus berezovskii, Bubalus bubalis, Bos javanicus, Bubalus carabanensis, Oryx dammah, and Dama dama—from the NCBI database. According to NCBI BLAST analysis, the sequence from Ovis aries showed identities of 100%, 100%, 99%, 99%, 99%, 99%, 99%, 98%, and 98% with the sequences of the other species (Figure 2).

**Figure 3:**
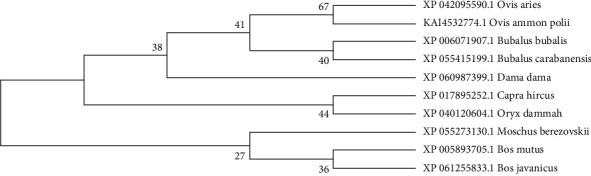
Phylogenetic tree based on the amino acid sequences of TGIF1 among different species.

**Figure 4:**
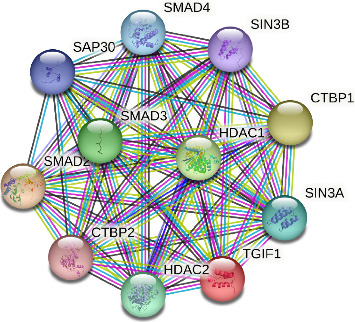
Prediction of the protein interaction networks of TGIF1 based on the STRING database.

The amino acid sequence alignment and phylogenetic analysis tree revealed that these eight TGIF1 species had a similar evolutionary pattern (Figures 2 and 3). The protein interaction network based on TGIF1 was analyzed using STRING v11. To explore the mechanisms through which TGIF1 may influence litter size in sheep, we analyzed protein interaction networks involving TGIF1 using the STRING database. Protein interaction networks indicated that TGIF1 interacts with known proteins that affect ovulation rate and LS, such as the SMAD family (SMAD2, SMAD3, and SMAD4) (Figure 4).

### 3.3. Genetic Parameter Analysis

The allelic, genotypic, and population genetic parameters (Ho, He, Ne, PIC, and HWE) of the nine SNPs in sheep TGIF1 are shown in Table 2. Seven of the nine SNPs were dominated by a normal allele. g.42725932 A>C exhibited the highest normal allele frequency (*A* = 0.64), whereas g.42725867 G>A exhibited the lowest frequency (*A* = 0.04). In contrast, g.42726042 A>C, g.42726052 A>G, and g.42726055 G>A shared a mutant allele frequency of 0.01, indicating a low prevalence of mutant alleles. Only two loci were in Hardy–Weinberg equilibrium (g. 42726009G>A and g. 42726042 A>C) (*p* > 0.05). Four sites had moderate polymorphism (0.25 < PIC < 0.5), indicating a moderate level of genetic diversity within TGIF1 in thin-tailed sheep.

### 3.4. Association of TGIF1 With Litter Size

Statistical analysis conducted using SPSS software revealed that none of the studied SNPs within the TGIF1 gene showed a statistically significant association with litter size in thin-tailed Indonesian sheep (*p* > 0.5) (Table 3). The absence of association may be linked to several variables, such as the restricted sample size, possible environmental impacts, or the polygenic characteristics of litter size. Although the SNPs found in this study did not exhibit significant relationships, they may still influence reproductive traits through interactions with other genes or environmental factors. Future research necessitates larger sample numbers and more extensive genetic investigations to investigate these possibilities.

### 3.5. Linkage Disequilibrium and Haplotype Frequency

**Figure 5:**
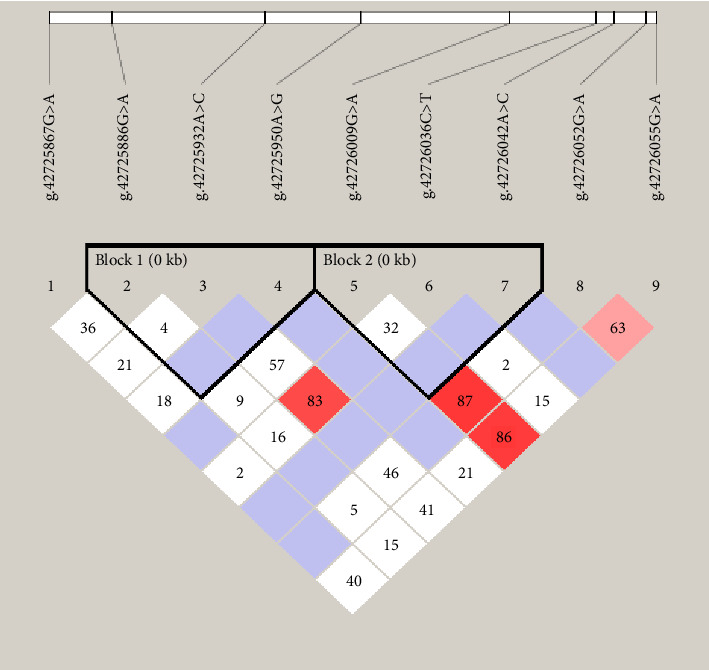
Disequilibrium of linkage among nine SNPS of TGIF1 in thin-tailed Indonesian sheep. The color of the square indicates the degree of linkage, the darker the color, the higher the degree of linkage. The value represents the strength of the correlation between sites (percentage). Haplotype blocks are indicated by dark lines.

To explore potential linkage patterns among variants within TGIF1, we conducted a comprehensive LD analysis using Haploview software, as depicted in Figure 5. Our findings revealed the presence of LD between six SNPs delineated into two distinct blocks: Block 1 comprised g.42725886 G> A, g.42725932 A > C, and g.42725950 A > G, while Block 2 encompassed g.42726009 G> A, g.42726036 C > T, and g.42726042 A > C within TGIF1. Within Block 1, *D*′ values ranged from 0.04 to 1.00, and corresponding *r*^2^ values ranged from 0.001 to 0.164. In Block 2, *D*′ values spanned from 0.324 to 1.00, with *r*^2^ values ranging from 0.001 to 0.011 (Table 4). The results indicate unique linkage patterns within the TGIF1 gene across the two blocks, with stronger LD observed in Block 1 compared with Block 2.

Employing a 1% haplotype frequency threshold in Haploview4.2, we identified a total of nine haplotypes across the six SNPs within the thin-tailed Indonesian sheep TGIF1 (Table 5). Examining haplotype frequencies, Block 1 exhibited H1 (AAA) as the most prevalent haplotype, with a frequency of 0.498. Conversely, H5 (GCA) exhibited the lowest frequency (0.072). In contrast, block 2 showed H6 (GCC) and H7 (GTC) as the most common haplotypes, with frequencies of 0.440 and 0.454, respectively. Notably, H8 (ATC) had the lowest frequency (0.032) (Table 5). The findings offer significant insights into haplotype diversity and LD structure within TGIF1, uncovering unique patterns of genetic variation in thin-tailed Indonesian sheep populations.

### 3.6. Analysis of the Association Between TGIF1 Gene Haplotypes and Litter Size

In thin-tailed Indonesian sheep, we conducted an analysis to assess the association between TGIF1 haplotypes and litter size (Table 6). The results show that there was no statistically significant association observed between haplotypes and litter size. This illustrates the idea that litter size is probably affected by both genetic and environmental variables, and the function of TGIF1 may be more complex than previously assumed.

## 4. Discussion

Small-scale farmers in Indonesia primarily rear thin-tailed sheep for meat production. Sheep are domesticated creatures that primarily fulfill human requirements by providing animal proteins for consumption. They possess metabolic and digestive characteristics that allow them to flourish under adverse conditions. SNPs from the candidate gene can be used as molecular markers for MAS if they show a strong link with litter size in certain sheep breeds. Using molecular indicators in a concentrated manner with MAS has the potential to improve the success of sheep selection by increasing litter size.

This study focused on analyzing the exon 3 of TGIF1 in sheep. Sequencing analysis confirmed these findings. The nucleotide sequence exhibited a similarity of 98%–100% to comparable genes found in goats, cattle, wild yaks, and deer. These findings suggest that TGIF1 orthologs exhibit a significant degree of commonality within the homeodomains of distantly related animals [23, 24]. TGIF1 lacks a signal peptide. The molecular weight of TGIF1 is 27,578.25, and its theoretical isoelectric point is 8.61. This prediction indicated that yak TGIF1 possesses 22 Ser, 3 Tyr, and 6 Thr phosphorylation sites. These protein characteristics resemble the findings from prior investigations conducted on other animal species [23, 25]. As TGIF1 engages with SMAD2 via protein–protein networks, a missense mutation is believed to diminish crucial protein-binding sites, resulting in a reduction in protein complex formation. Subsequently, with a decline in the protein complex, the TGIF1–SMAD system persists in binding to promoters or enhancers.

Nine SNPs were identified in sheep, including six novel SNPs: g.42725867 G>A, g.42725950 A/G, g.42726009 G/A, g.42726036 C/T, g.42726052 G/A, and g.42726055 G/A. Among these, g.42726055 G>A resulted in a nonconserved amino acid change (Glu217Lys) and g.42725932 A>C resulted in Thr176Pro. Both substitutions were predicted to be deleterious, suggesting potential functional implications for these novel nonsynonymous SNPs, with an expected negative impact on protein function. The identification of previously unreported variants contributes to our understanding of the genetic diversity within the studied sheep population. In addition, a range of TGIF1 SNPs were found in exon 3. TGIF1, like other components of the TGF-β signaling pathway, exerts its biological effects following interaction with specific receptors, similar to how TGF-β, activin/nodal, and BMP pathways utilize distinct receptor combinations to activate corresponding SMAD proteins and downstream signaling cascades. However, the analysis results of SIFT, PolyPhen-2, and PROVEAN indicated that the effects of the five nonsynonymous mutations on the structure and function of the TGIF1 protein were benign [26–28], suggesting that these loci may influence sheep litter size through other regulatory mechanisms rather than directly affecting TGIF1 protein function. Further research will be needed to clarify the mechanism of their action. This persistence hinders TGIF1's effective inhibition of the SMAD complex, compromising its capacity to regulate TGF-β signaling [17].

Five of the SNPs showed low polymorphism (PIC ≤ 0.25) and four exhibited moderate polymorphisms, which suggest these loci may have limited genetic diversity and thus could be less effective in detecting broader genetic variation within the population. However, SNPs with moderate polymorphism could still be useful as markers for genetic selection, particularly in traits like litter size, as they can potentially capture sufficient genetic variance for selection purposes. It is important to note that the observed polymorphism levels may be influenced by the sample size. Deviations from Hardy–Weinberg equilibrium were observed in SNPs (SNP1, SNP2, SNP3, SNP5, SNP6, SNP8, and SNP9), whereas SNP4 and SNP7 remained consistent with equilibrium. This suggests that factors like prolonged artificial or natural selection, genetic migration, and drift might be influencing the population [29].

Zhang et al. [15] investigated the function of TGIF1 in reproductive processes. However, its impact on litter size remains unexplored. In the present study, we used association analysis to investigate the relationship between TGIF1 polymorphisms and litter size in thin-tailed Indonesian sheep. TGIF1 locus SNP7 (rs1092900782) showed a synonymous variation in a previous study [12]. This variant has been found in six different breeds. Consistent with a previous study, SNP rs1092900782 was associated with two genotypes, CC and CA. Interestingly, ewes with the CA genotype had a significantly larger litter size than those with the wild-type CC genotype. These findings indicated that this synonymous mutation may have detrimental effects.

TGIF1 exerts a negative regulatory effect on TGF-β-activated genes, specifically SMAD2 and SMAD4 [12, 13, 17, 23] (Wijayanti et al., 2022a and Wijayanti et al., 2022b), and TGF-β/SMAD signaling plays crucial roles in mediating reproduction [15, 30]. Disruption of TGF-β/SMAD signaling has been linked to various reproductive issues, including sterility [31]. The interaction of the TGIF1 gene with SMAD proteins affects follicular development and ovarian function, which are essential for fertility. Considering that SMAD2, a crucial element of TGF-β signaling, significantly influences oocyte maturation and follicular development (Wijayanti et al., 2022a), alterations in TGIF1 may affect reproductive characteristics beyond litter size. The GDF-9 protein is involved in regulating the abundance of SMAD2/3 protein and has inhibitory actions on FSH-induced E 2 production [32]. In pigs, suppression of endogenous SMAD4 decreases estradiol synthesis in GCs [33]. Interestingly, in goose GCs, lower levels of SMAD9 mRNA correlate with higher concentrations of estradiol and progesterone, while progesterone concentrations remain unaffected [34]. Although TGIF1 SNPs were not significantly associated with litter size in thin-tailed Indonesian sheep, they may still influence other reproductive traits, such as follicular development, ovulation rate, or hormone production, through mechanisms involving epigenetic regulation or interactions with other genes. Specifically, the interaction between TGIF1 and HDAC1, as revealed by STRING data, suggests that TGIF1 may modulate histone acetylation and gene expression in GCs, thereby affecting follicle development and ovulation. In addition, TGIF1's role in TGF-β signaling and its interactions with SMAD proteins could further contribute to reproductive efficiency in ways not captured by litter size alone. Future studies should investigate these potential mechanisms to clarify the broader relevance of TGIF1 SNPs in reproductive biology.

Clinical investigations have provided evidence indicating that abnormalities or genetic variations in TGF-β-mediated signaling are associated with various reproductive issues, including polycystic ovary syndrome, ovulation failure, and ovarian malignancies [30]. Given TGIF1's role in negatively regulating SMAD2/3 phosphorylation and its impact on steroidogenesis in GCs, it is plausible that similar mechanisms in sheep could modulate ovarian function and influence litter size by affecting follicular development and hormone production [13]. Hence, although the insufficient number of ewes may explain our inability to identify the third genotype at the SNP7 locus or establish connections between these loci, we propose that the lack of a homozygous AA genotype at the SNP7 locus in TGIF1 is linked to variations in litter size in thin-tailed Indonesian sheep. This association is likely attributable to the modified function of TGIF1, which affects reproductive characteristics. Similar roles of TGIF1 have been found in other species. In humans, TGIF1 regulates TGF-β signaling via SMAD proteins, impacting ovarian function and steroidogenesis [35, 36]. TGIF1 regulates GC steroidogenesis and follicular growth in cattle, as in sheep [32, 33]. In yaks, TGIF1 affects cell survival and steroid hormone synthesis, which is essential for reproduction [23]. Studies across species indicate TGIF1's conserved role in TGF-β signaling, suggesting its modulation may be crucial for regulating fertility and reproductive features.

The lack of significant associations between SNPs and litter size may be due to the polygenic nature of the trait, involving complex interactions among genes such as TGIF1, which influences SMAD signaling and steroidogenesis. Other factors, such as epistatic interactions and post-transcriptional regulation, may also play a role. In addition, the limited sample size may have reduced the statistical power to detect subtle effects. Future studies with larger, more diverse datasets and consideration of gene–gene and gene–environment interactions are needed to better understand the genetic basis of litter size in this breed.

The investigation revealed six SNPs within TGIF1, demonstrating LD in thin-tailed ewes. These SNPs were categorized into two distinct blocks (Table 4). To the best of our knowledge, this study represents the first examination of LD among these SNPs within TGIF1 in sheep. If these genetic variations are correlated with genes that influence differences in reproductive characteristics, the categorization of individuals based on marker alleles is anticipated to yield discernible variations in physical attributes. Consequently, it is imperative to consider the phenomenon of LD when incorporating this gene polymorphism into the breeding regimen of sheep with augmented litter size. The distribution of haplotypes encapsulates the ancestral structure, rendering them more influential than individual SNPs in elucidating significant connections with major traits [37].

Previous studies indicated that variations within a specific gene can affect traits through LD [38]. The functional capacity of a gene and the probability of harboring pathogenic gene variants are determinants that affect the strength or weakness of the LD [20, 36]. A single mutation can exert a substantial influence on the functioning of genes and their characteristics, whereas the recombination of distinct mutation loci plays a crucial role in the emergence of genetic and trait diversity [16].

In general, sheep exhibit relatively lower levels of LD compared with other domestic species, such as dairy cattle. LD in sheep typically spans shorter genomic distances, reflecting their population history, including selection pressures and effective population size [40, 41]. This reduced LD limits the effectiveness of individual markers for MAS, as fewer loci are in strong association with causative genes. Consequently, achieving sufficient power for genetic association studies in sheep often requires higher-density genetic marker panels.

The current investigation observed no LD between SNPs in either block locus. The LD values for Blocks 1 (D 1; *r*^2^ = 0.164) and 2 (D 1; *r*^2^ = 0.011) were determined. Based on these discoveries, we can affirm that these two mutations have a limited association; however, further investigation is necessary to ascertain the precise molecular mechanisms underlying their effects. Haplotypes are superior to SNPs as genetic markers because of their increased likelihood of being inherited in conjunction. Haplotypes showed greater efficacy than did SNPs for variables with moderate-to-low heritability [42, 43]. Although this study identified haplotypes with no statistically significant association with litter size, the observed trends provide valuable insights for future research. Specifically, H3 in Block 1 was associated with a higher average litter size compared with other haplotypes within the same block, and H6 in Block 2 exhibited a larger litter size relative to H7–H9. These findings, while not statistically significant, suggest that certain haplotypes may influence reproductive performance.

Thus, thin-tailed Indonesian sheep selection strategies to increase litter size may use H3 and H6 as preliminary genetic indicators. To prove their usefulness in MAS, higher sample sizes and functional studies are needed. Increased marker density, sample size, could improve sheep reproductive trait MAS resolution and reliability. Higher sample size may help explain the lack of the genetic association.

## 5. Conclusion

Nine polymorphic sites were found, five of which caused amino acid changes. These genetic variants indicate a possible impact on litter size in Indonesian thin-tailed sheep. The lack of significant associations limits the applicability of the results. The limited sample size and emphasis on a singular sheep community underscore the necessity for additional validation in larger, diverse populations.

## Figures and Tables

**Table 1:** Identification code of the variant, position in the current assembly, amino acid changes, and sorting intolerant from tolerant (SIFT) score (prediction of the effect of an amino acid change on protein function).

SNP number	Oar 4.0 (XM_042239656.2)	DbSNPs	CDS position	Amino acids
SNP1	g.42725867 G/A		461	R154K AGG/AAG
SNP2	g.42725886 G/A	rs1086957063	480	P160P CCG/CCA
SNP3	g.42725932 A/C	rs415585940	526	T176P ACC/CCC
SNP4	g.42725950 A/G		544	K182E AAA/GAA
SNP5	g.42726009 G/A		603	Q201Q CAG/CAA
SNP6	g.42726036 C/T		630	D210D GAC/GAT
SNP7	g.42726042 A/C	rs1092900782	636	S212S TCA/TCC
SNP8	g.42726052 G/A		646	A216T GCA/ACA
SNP9	g.42726055 G/A		649	E217K GAG/AAG

**Table 2:** Frequencies of alleles and genotypes of the TGIF1 gene in thin-tailed sheep.

SNP number	Locus	Genotype			Allele frequncy		He	PIC	Ne	Chi square test (*p* value)
SNP1	g. 42725867 G>A	AA (2)	AG (0)	GG (45)	A 0.04	G 0.96	0.081	0.076	1.09	*p* < 0.05
SNP2	g. 42725886 G>A	AA (33)	AG (8)	GG (6)	A 0.79	G 0.21	0.335	0.331	1.50	*p* < 0.05
SNP3	g. 42725932 A>C	AA (14)	AC (33)	CC (0)	A 0.65	C 0.35	0.460	0.474	1.86	*p* < 0.05
SNP4	g. 42725950 A>G	AA (39)	AG (8)	GG (0)	A 0.91	G 0.09	0.156	0.163	1.18	*p* > 0.05
SNP5	g. 42726009 G>A	GG (41)	GA (3)	AA (3)	G 0.90	A 0.10	0.173	0.18	1.20	*p* < 0.05
SNP6	g.42726036 C>T	CC (2)	CT (44)	TT (1)	C 0.53	T 0.47	0.510	0.449	1.99	*p* < 0.05
SNP7	g. 42726042 A>C	AA (0)	AC (1)	CC (46)	A 0.01	C 0.99	0.021	0.019	1.02	*p* > 0.05
SNP8	g. 42726051 A>G	AA (4)	AG (2)	GG (41)	A 0.11	G 0.89	0.19	0.195	1.23	*p* < 0.05
SNP9	g. 42726059 G>A	GG (38)	GA (3)	AA (6)	G 0.84	A 0.16	0.268	0.268	1.37	*p* < 0.05

*Note:* Ho, homozygosity; He, heterozygosity; Ne, effective allele numbers.

Abbreviations: HWE: Hardy–Weinberg equilibrium; PIC: polymorphism information content.

**Table 3:** Least squares mean and standard error for litter size of different genotypes of the TGIF1 gene in thin-tailed sheep.

Locus	Genotype (*n*)	LS
g.42725867 G/A	AA (*n* = 2)	1.50 ± 0.707
	GG (*n* = 45)	1.91 ± 0.668

g.42725886 G/A	AA (*n* = 33)	1.85 ± 0.817
	AC (*n* = 8)	1.50 ± 0.535
	CC (*n* = 6)	2.17 ± 0.408

g.42725932 A/C	AA (*n* = 14)	1.93 ± 0.616
	AC (*n* = 33)	1.79 ± 0.696
	CC (*n* = 0)	—

g.42725950 A/G	AA (*n* = 39)	1.90 ± 0.598
	AG (*n* = 8)	1.88 ± 0.991

g.42726009 G/A	AA (*n* = 3)	1.67 ± 0.577
	GA (*n* = 3)	1.67 ± 0.577
	GG (*n* = 41)	1.93 ± 0.685

g.42726036 C/T	CC (*n* = 2)	2.50 ± 0.707
	CT (*n* = 44)	1.82 ± 0.657
	TT (*n* = 1)	1.00 ± 0.000

g.42726042 A/C	CA (*n* = 1)	2.00 ± 0.000
	CC (*n* = 46)	1.89 ± 0.674

g.42726052 G/A	AA (*n* = 4)	1.50 ± 0.577
	AG (*n* = 2)	1.50 ± 0.707
	GG (*n* = 41)	1.95 ± 0.669

g.42726055 G/A	AA (*n* = 6)	1.83 ± 0.408
	AG (*n* = 3)	2.00 ± 1.000
	GG (*n* = 38)	1.89 ± 0.689

*Note: n*, number of samples.

Abbreviations: LS, litter size.

**Table 4:** Estimated linkage disequilibrium for the SNPs identified in the TGIF1 gene.

	*D*′	*r* ^2^
*Block 1*		
g.42725886/g.42725932	0.04	0.001
g.42725886/g.42725950	1	0.025
g.42725932/g.42725950	1	0.164

*Block 2*		
g.42726009/g.42726036	0.324	0.011
g.42726009/g.42726042	1	0.001
g.42726036/g.42726042	1	0.011

**Table 5:** Estimates of haplotypes frequencies observed in the TGIF1 gene.

Haplotype	g.42725886 G>A	g.42725932 A>C	g.42725950 A>G	Haplotype frequency
*Block 1*				
H1 (AAA)	A	A	A	0.498
H2 (ACA)	A	C	A	0.205
H3 (GAA)	G	A	A	0.141
H4 (ACG)	A	C	G	0.085
H5 (GCA)	G	C	A	0.072

**Haplotype**	**g.42726009 G>A**	**g.42726036 C>T**	**g.42726042 A>C**	**Haplotype frequency**

*Block 2*				
H6 (GCC)	G	C	C	0.440
H7 (GTC)	G	T	C	0.454
H8 (ATC)	A	T	C	0.032
H9 (ACC)	A	C	C	0.064

**Table 6:** TGIF1 haplotypes association analysis and litter size in thin-tailed Indonesian sheep.

Haplotype	Number of samples	Litter size (mean ± SD)	*p* value
*Block 1*			
H1 (AAA)	41	1.78 ± 0.690	0.971
H2 (ACA)	30	1.77 ± 0.728	
H3 (GAA)	13	1.92 ± 0.641	
H4 (ACG)	8	1.75 ± 1.035	
H5 (GCA)	9	1.78 ± 0.667	

*Block 2*			
H6 (GCC)	43	1.86 ± 0.675	0.915
H7 (GTC)	41	1.83 ± 0.677	
H8 (ATC)	5	1.80 ± 0.477	
H9 (ACC)	6	1.67 ± 0.516	

## Data Availability

The data supporting the conclusions of this research are available from the corresponding author upon reasonable request.

## Supporting Information

Supporting Figure S1: The supporting information include chromatograms from sanger sequencing results, providing visual confirmation of nucleotide sequences and variant positions.
