# Supplementary material for: Improved Litter Size in Thin-Tailed Indonesian Sheep Through Analysis of TGIF1 Gene Polymorphisms
Source: Vet Med Int. 2025 Apr 21;2025:7778088. doi: 10.1155/vmi/7778088 (PMC12037246; doi:10.1155/vmi/7778088)
Supplement: Supporting Information — Additional supporting information can be found online in the Supporting Information section. [file 7778088.f1.pdf]

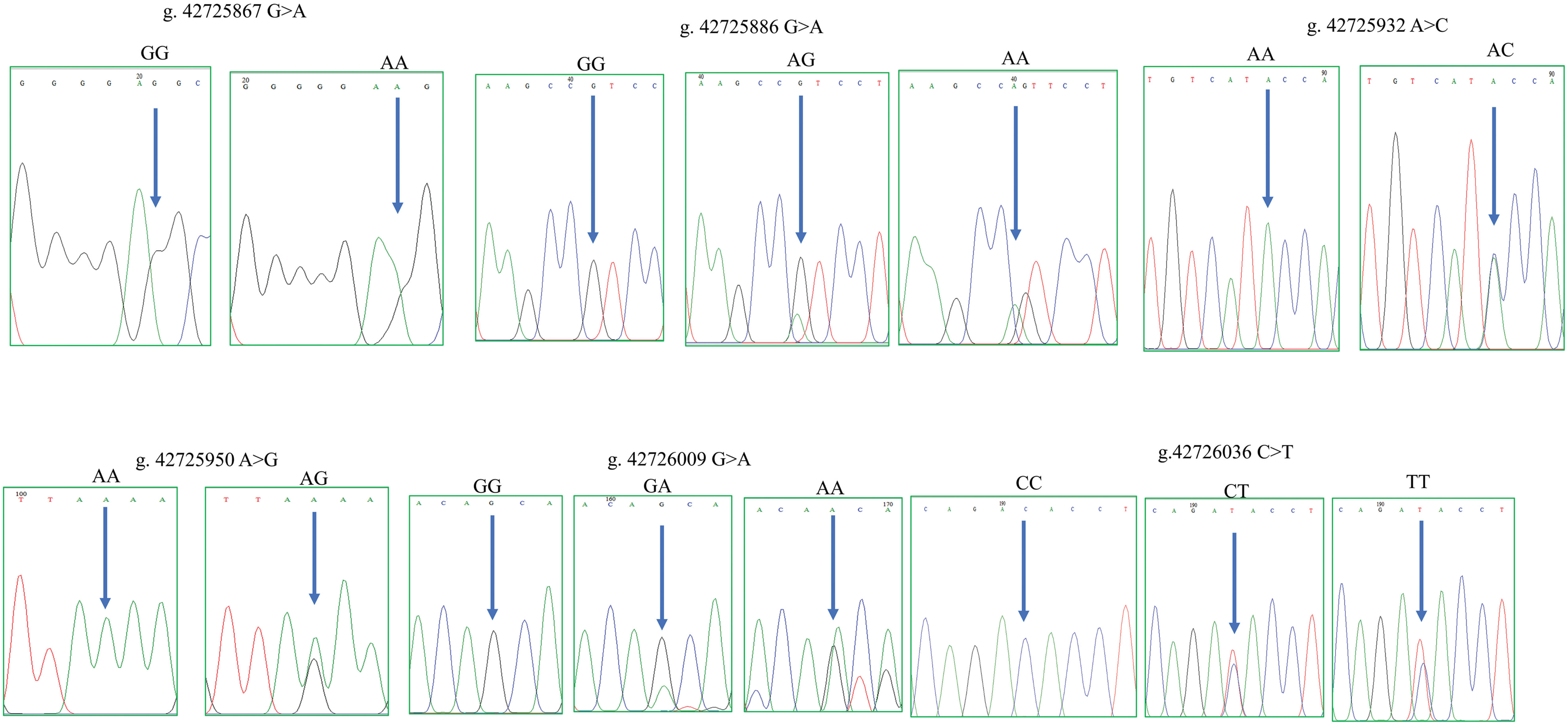

g. 42725867 G>A
GG
AA
g. 42725886 G>A
GG
AG
AA
g. 42725932 A>C
AA
AC
g. 42725950 A>G
AA
AG
g. 42726009 G>A
GG
GA
AA
g.42726036 C>T
CC
CT
TT

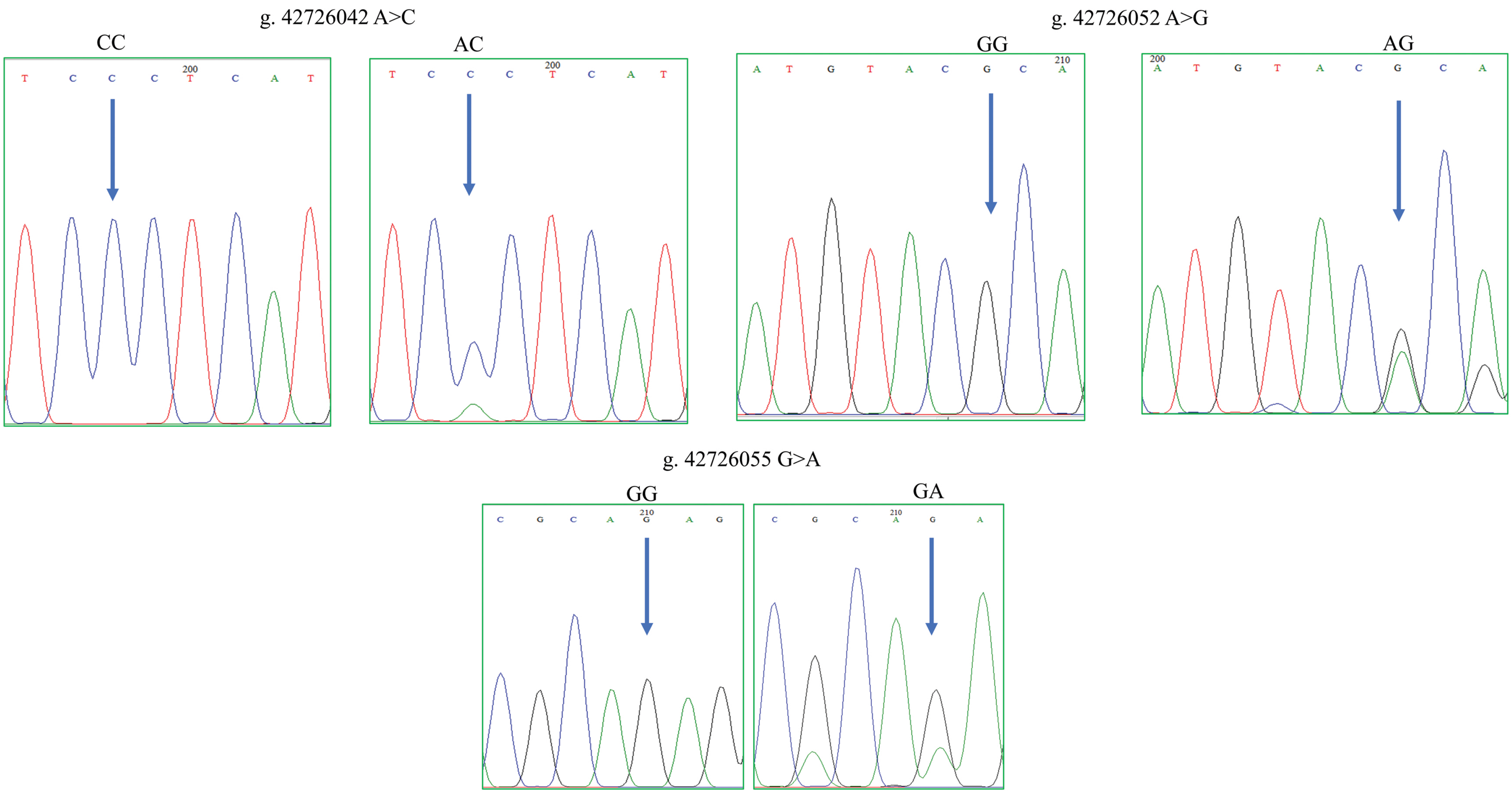

Figure S1. Identified SNP in the exon 3 of TGIF1 gene in Indonesian thin-tailed sheep
